# Thrombocytopenia as a Bleeding Risk Factor in Atrial Fibrillation and Coronary Artery Disease: Insights From the AFIRE Study

**DOI:** 10.1161/JAHA.123.031096

**Published:** 2023-10-17

**Authors:** Raisuke Iijima, Masahide Tokue, Masato Nakamura, Satoshi Yasuda, Koichi Kaikita, Masaharu Akao, Junya Ako, Tetsuya Matoba, Katsumi Miyauchi, Nobuhisa Hagiwara, Kazuo Kimura, Atsushi Hirayama, Kunihiko Matsui, Hisao Ogawa, the AFIRE Investigators

**Affiliations:** ^1^ Division of Cardiovascular Medicine Toho University Ohashi Medical Center Tokyo Japan; ^2^ Kawasaki Miyamaedaira Tokue Internal‐Cardiovascular Medical Clinic Kawasaki Japan; ^3^ Department of Cardiovascular Medicine Tohoku University Graduate School of Medicine Sendai Japan; ^4^ Division of Cardiovascular Medicine and Nephrology, Department of Internal Medicine, Faculty of Medicine University of Miyazaki Miyazaki Japan; ^5^ Department of Cardiology National Hospital Organization Kyoto Medical Center Kyoto Japan; ^6^ Department of Cardiovascular Medicine Kitasato University School of Medicine Sagamihara Japan; ^7^ Department of Cardiovascular Medicine, Faculty of Medical Sciences Kyushu University Fukuoka Japan; ^8^ Department of Cardiovascular Medicine Juntendo Tokyo Koto Geriatric Medical Center Tokyo Japan; ^9^ Department of Cardiology Tokyo Women’s Medical University Tokyo Japan; ^10^ Department of Cardiology Yokosuka City Hospital Yokosuka Japan; ^11^ Department of Cardiology Osaka Police Hospital Osaka Japan; ^12^ Department of General Medicine Kumamoto University Hospital Kumamoto Japan; ^13^ Kumamoto University Kumamoto Japan

**Keywords:** atrial fibrillation, chronic coronary syndrome, thrombocytopenia, Anticoagulants, Percutaneous Coronary Intervention, Atrial Fibrillation

## Abstract

**Background:**

Thrombocytopenia poses a risk of bleeding in patients with chronic coronary syndrome after coronary intervention. However, whether thrombocytopenia also increases the bleeding risk in patients with atrial fibrillation and chronic coronary syndrome remains unclear.

**Methods and Results:**

This study evaluated the AFIRE (Atrial Fibrillation and Ischemic Events With Rivaroxaban in Patients With Stable Coronary Artery Disease) trial. Thrombocytopenia was defined as platelet count <100 000/mm^3^ level at enrollment. Primary end points included incidence of major bleeding based on the International Society on Thrombosis and Hemostasis criterion and major adverse cardiovascular ischemic events (cardiac death, myocardial infarction, and stroke). A total of 2133 patients were classified into the thrombocytopenia (n=70) and nonthrombocytopenia (n=2063) groups. Major bleeding was significantly higher in the thrombocytopenia group than in the nonthrombocytopenia group (10.0% versus 4.1%, *P*=0.027). The thrombocytopenia group tended to have a higher risk of major adverse cardiovascular ischemic events (11.4% versus 6.2%, *P*=0.08). The bleeding incidence was significantly higher in patients with thrombocytopenia receiving combination therapy with rivaroxaban and a single antiplatelet drug (thrombocytopenia group, 14.3%, versus nonthrombocytopenia group, 5.0%; hazard ratio, 3.18 [95% CI, 1.27–7.97], *P*=0.014). Thrombocytopenia was an independent predictor of major bleeding (hazard ratio, 2.57 [95% CI, 1.19–5.56], *P*=0.017).

**Conclusions:**

Among patients with atrial fibrillation and chronic coronary syndrome, thrombocytopenia was significantly associated with increased risk of major bleeding. Selecting drugs for patients with thrombocytopenia continuing antithrombotic therapy should be given special consideration.

**Registration:**

URL: https://www.clinicaltrials.gov; Unique identifier: NCT02642419. https://www.umin.ac.jp/ctr/; Unique identifier: UMIN000016612.

**Nonstandard Abbreviations and Acronyms**
AFIRE: Atrial Fibrillation and Ischemic Events With Rivaroxaban in Patients With Stable Coronary Artery Disease
CCS: chronic coronary syndrome
MACE: major adverse cardiovascular ischemic events

**Clinical Perspective**

**What Is New?**

- Although guidelines currently recommend antithrombotic monotherapy for patients with chronic coronary syndrome (CCS) and atrial fibrillation (AF), whether this recommendation should be extended to patients with thrombocytopenia with CCS and AF remains unclear.
- This study is the first to demonstrate favorable outcomes of rivaroxaban monotherapy in thrombocytopenia patients with CCS and AF.

**What Are the Clinical Implications?**

- The findings of this study could serve as a reference for clinicians in planning approaches for antithrombotic therapy in patients with thrombocytopenia with CCS and AF.
- To further investigate the efficacy and safety of earlier de‐escalation to rivaroxaban monotherapy after coronary intervention for patients with AF and CCS, well‐designed, randomized controlled trials are necessary.

Thrombocytopenia is rare but is an important risk factor for bleeding and ischemic complications in patients receiving drug‐eluting stent and dual antiplatelet therapy.^1^, ^2^, ^3^ However, only few studies have investigated the relationship between thrombocytopenia and clinical events because most randomized trials did not consider thrombocytopenia in the inclusion criteria. Indeed, patients with atrial fibrillation (AF) with platelet count <100 000/mm^3^ or <90 000/mm^3^ were excluded in landmark large‐scale randomized trials that evaluated the safety of direct oral anticoagulants.^4^, ^5^, ^6^, ^7^ The HORIZONS‐AMI randomized trial (harmonizing outcomes with revascularization and stents in acute myocardial infarction)^8^ showed that thrombocytopenia is associated with 30‐day cardiac death and major bleeding in ST‐segment–elevation acute myocardial infarction. However, patients with severe thrombocytopenia with platelet count of <100 000/mm^3^ were excluded based on its study protocol. With that background, reports from CREDO‐Kyoto PCI/CABG registry cohort‐2 and the Nationwide Inpatient Sample database provide evidence that the presence of thrombocytopenia is associated with major bleeding and mortality risks.^9^, ^10^ Given the limited number of observational studies examining the safety and efficacy of direct oral anticoagulant in patients with AF with lower platelet counts, how thrombocytopenia affects bleeding complications and mortality of patients with AF remains unclear.^11^, ^12^ Moreover, the optimal antithrombotic management including the combination of antiplatelet and direct oral anticoagulant agents in patients with AF and concurrent thrombocytopenia is still unclear. The recent European Hematology Association Guideline recommends that patients with thrombocytopenia and bleeding risk should use the lowest effective anticoagulant dose if antiplatelet agents and direct oral anticoagulants need to continue. Furthermore, only low‐dose aspirin should be maintained if the platelet count is <50 000/mm^3^, because the combination of antiplatelet agent and oral anticoagulant is not recommended.^13^

The AFIRE (Atrial Fibrillation and Ischemic Events With Rivaroxaban in Patients With Stable Coronary Artery Disease) trial included patients diagnosed with AF and chronic coronary syndromes (CCS) >1 year after revascularization or those with angiographically confirmed coronary artery disease not requiring revascularization.^14^ This trial demonstrated that rivaroxaban monotherapy was noninferior to combination therapy using rivaroxaban and antiplatelet therapy as regards cardiovascular events and superior against major bleeding in CCS‐complicated AF.

This post hoc analysis of the AFIRE trial aimed to investigate the association between thrombocytopenia with an increased risk of major bleeding and cardiovascular events in patients with both AF and CCS. The relationship between the presence or absence of thrombocytopenia and the antithrombotic regimen on the clinical outcome of patients has also been investigated.

## METHODS

### Study Population

The design and results of the AFIRE trial have been reported previously.^14^ Briefly, the trial was a multicenter, prospective, randomized, open‐label, parallel trial including 2236 patients diagnosed with AF and CCS and treated with antithrombotic and antiplatelet therapy for at least 1 year. Study participants were randomly assigned in a 1:1 ratio to receive either rivaroxaban monotherapy (10 mg once daily for patients with a creatinine clearance of 15 to 49 mL per minute or 15 mg once daily for patients with a creatinine clearance of ≥50 mL per minute) or combination therapy with rivaroxaban at the previously stated doses plus an antiplatelet agent (either aspirin or a P2Y_12_ inhibitor, as per the treating physician's discretion). The primary efficacy analysis was based on the modified intention‐to‐treat approach. The study population included 2215 patients who had undergone randomization after 21 patients were excluded due to technical reasons, such as incomplete registration and withdrawal of consent for data use, for not participating in the trial.^14^ Fewer key exclusion criteria were another aspect of the trial: history of stent thrombosis, coexisting active tumor, and poorly controlled hypertension. Furthermore, the AFIRE trial was terminated earlier than planned on the recommendation of the Independent Data and Safety Monitoring Committee due to an increased risk of death from any cause in the combination therapy group.

The present study analyzed 2215 patients from the modified intention‐to‐treat population of the AFIRE trial and evaluated 2133 patients for whom platelet count data were available. In this study, units of all platelet counts measured at each hospital were standardized to “/mm^3^,” and thrombocytopenia was defined by a platelet count <100 000/mm^3^ level at enrollment according to the consensus document for defining patients with high bleeding risk.^15^ Finally, the thrombocytopenia group included 70 patients (3.3%). Figure S1 shows the distribution of baseline platelet counts, with a median of 18.5 x 10^4^/mm^3^.

### Clinical Outcomes

The incidence of major bleeding and major adverse cardiovascular ischemic events (MACE) were the primary end points of this study. Major bleeding was defined based on the criteria of the International Society on Thrombosis and Hemostasis.^16^ MACE was defined as a composite of cardiovascular death, myocardial infarction, or stroke.^17^ The secondary end points, individual components, and all causes of death were also assessed.^18^ An independent clinical events committee performed a blinded evaluation of the outcomes.

The study was approved by the local ethics committee at each participating center and complied with the tenets of the Declaration of Helsinki. A written informed consent was obtained from all patients before any participation. Data supporting the study findings are available from the corresponding author upon reasonable request.

### Statistical Analysis

Data are presented as mean±(SD) or percentages. For continuous variables, differences between groups were assessed using unpaired 2‐tailed Student *t* tests, and for categorical variables, the χ^2^ test or the Fisher exact test was used. In this study, time‐to‐first‐event analysis for major bleeding events and the first event analysis for MACE were both performed. To evaluate event‐rate difference with or without thrombocytopenia and between rivaroxaban monotherapy and combined therapy, Kaplan–Meier survival curves and the log‐rank test were performed. Moreover, to assess the independent correlates of major bleeding and MACE, multivariable Cox proportional hazard models were used. The Fine–Gray hazard model was also used to accurately evaluate the risk of major bleeding event because the initial bleeding event and death from any cause were considered as competing risks. All variables are compared in Tables S1 and S2. Eventually, adjustment was performed to calculate hazard ratios (HR) and 95% CIs using only clinically relevant variables with *P* values of <0.1 in the univariable analysis. The restricted cubic spline regression model was used to assess the potentially nonlinear association between platelet counts and major bleeding or MACE. All analyses were performed using the R package. A *P* value of <0.05 was considered statistically significant.

## RESULTS

### Patient Characteristics

Table 1 shows the baseline patient characteristics of the thrombocytopenia (n=70) and nonthrombocytopenia groups (n=2063). Compared with the nonthrombocytopenia group, the thrombocytopenia group included older patients, a greater likelihood of diabetes and history of heart failure, and had a lower proportion of women. No significant difference was observed in the hemoglobin level and glomerular filtration rate between groups. Regarding medication, all patients were treated with an appropriate dose of rivaroxaban, and no significant difference was found in the types of antiplatelet drugs used between the thrombocytopenia and nonthrombocytopenia groups (aspirin, 37.1% versus 35.9%, *P*=0.90; P2Y_12_ receptor antagonists, 12.9% versus 14.1%, *P*=0.94).

**Table 1:** Baseline Characteristics of Patients With Atrial Fibrillation With or Without Thrombocytopenia

	Thrombocytopenia (+)	Thrombocytopenia (−)	*P* value
	n=70 (%)	n=2063 (%)	
Age, y	76.3±7.6	74.3±8.2	0.045
Female sex	4 (5.7)	444 (21.5)	0.0005
Body mass index, kg/m^2^	24.2±2.5	24.5±3.7	0.58
Hypertension	59 (84.3)	1765 (85.6)	0.73
Systolic blood pressure, mm Hg	125.1±17.4	126.3±15.9	0.57
Dyslipidemia	44 (62.9)	1441 (69.8)	0.23
Diabetes	40 (57.1)	855 (41.4)	0.01
Current smoker	8 (11.4)	269 (13.0)	0.86
Chronic heart failure	36 (51.4)	726 (35.2)	0.007
e‐GFR, mL/min per 1.73 m^2^	56.9±19.5	58.1±16.2	0.54
Hemoglobin, g/dL	13.3±1.9	13.5±1.7	0.29
CHADS2 score	2.5±1.3	2.5±1.2	0.82
HAS‐BLED score	2.2±0.8	2.1±0.8	0.37
Prior myocardial infarction	26 (37.1)	718 (34.8)	0.70
Prior stroke	7 (10.0)	303 (14.7)	0.39
Prior PCI	52 (74.3)	1455 (70.5)	0.59
Prior CABG	10 (14.3)	229 (11.1)	0.44
Polyvascular disease	36 (51.4)	1018 (49.3)	0.82
Treatment assignment			
Rivaroxaban monotherapy	35 (50.0)	1040 (50.4)	0.99
Combination therapy	35 (50.0)	1023 (49.6)	
Medications			
Aspirin	26 (37.1)	740 (35.9)	0.90
P2Y_12_ receptor antagonists	9 (12.9)	291 (14.1)	0.94
Proton pump inhibitors	41 (58.6)	1273 (61.7)	0.62
Multivessel coronary disease	18 (25.7)	347 (16.8)	0.07
Left anterior descending artery	28 (40.0)	859 (41.6)	0.81
Left circumflex artery	20 (28.6)	400 (19.4)	0.07
Left main coronary artery	2 (2.9)	43 (2.1)	0.66
Right coronary artery	25 (35.7)	550 (26.7)	0.10

Continuous variables are expressed as the mean±SD. Categorical variables are expressed as n (percentage). Combination therapy=treatment with rivaroxaban plus antiplatelet agent. CABG indicates coronary artery bypass graft surgery; eGFR, estimated glomerular filtration rate; and PCI, percutaneous coronary intervention.

### Thrombocytopenia and Clinical Outcomes

The mean of clinical follow‐up was 693.6±268.3 days. Table 2 shows that the thrombocytopenia group has a higher incidence of major bleeding than the nonthrombocytopenia group (10.0% versus 4.1%). Regarding thrombotic events, the composite incidence of cardiovascular death, myocardial infarction, or stroke was almost twice that of the thrombocytopenia group, although no significant statistical difference was observed (11.4% versus 6.2%, *P*=0.08). The frequency of all‐cause death was significantly higher in the thrombocytopenia group than that in the nonthrombocytopenia group, mainly due to noncardiovascular death. Even when platelet counts <150 000/mm^3^ were analyzed, a similar trend was observed (Table S3).

**Table 2:** Clinical Outcomes of Patients With Atrial Fibrillation With or Without Thrombocytopenia

	Thrombocytopenia (+)	Thrombocytopenia (−)	*P* value
	n=70 (%)	n=2063 (%)	
Primary end points			
Major bleeding	7 (10.0)	84 (4.1)	0.027
Cardiovascular death+myocardial infarction+stroke	8 (11.4)	127 (6.2)	0.08
Secondary end points			
All cause death	12 (17.1)	100 (4.8)	0.0002
Cardiovascular death	4 (5.7)	64 (3.1)	0.26
Myocardial infarction	1 (1.4)	19 (0.9)	0.49
Stroke	3 (4.3)	60 (2.9)	0.46

**Figure 1:**
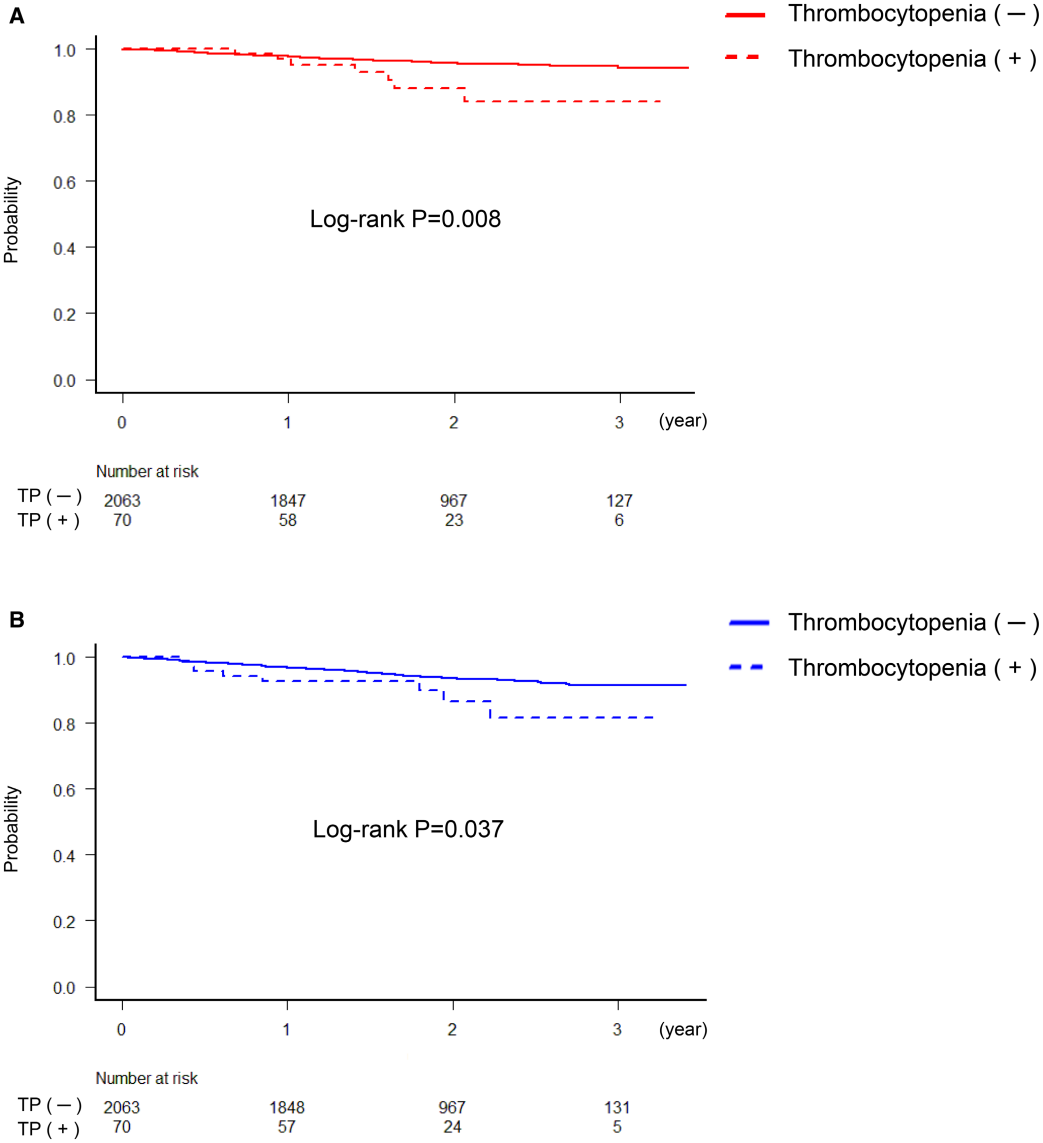
Kaplan–Meier curves for major bleeding and MACE. **A**, Kaplan–Meier estimates of survival with freedom from major bleeding with and without thrombocytopenia. **B**, Kaplan–Meier estimates of survival with freedom from MACE with and without thrombocytopenia. MACE indicates major adverse cardiovascular ischemic events.

**Figure 2:**
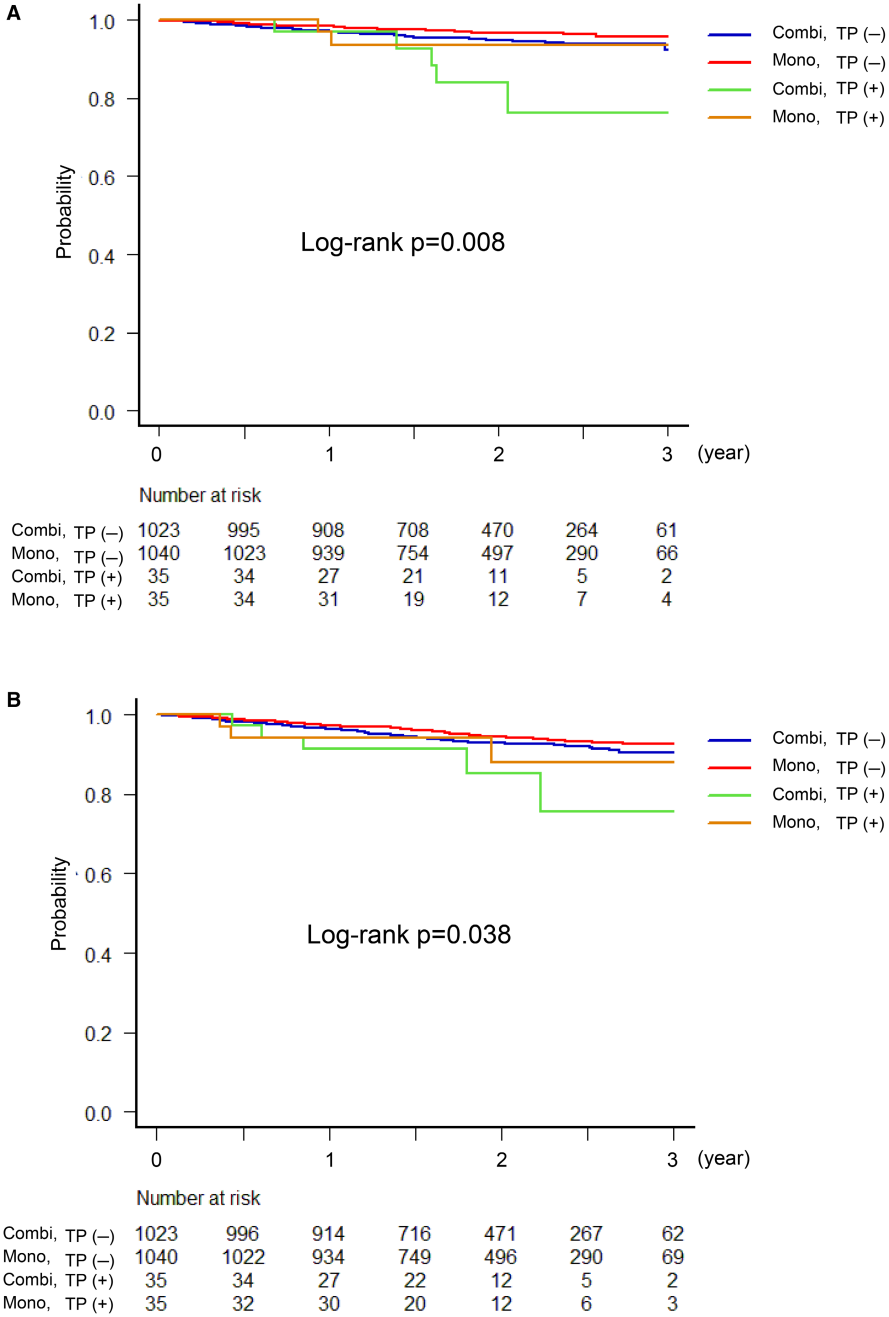
Kaplan–Meier curves for thrombocytopenia and treatment group. Kaplan–Meier curves for (**A**) major bleeding and (**B**) MACE (4 groups, based on the thrombocytopenia/nonthrombocytopenia group and treatment type). Combi indicates combination therapy with rivaroxaban plus antiplatelet agent; MACE, major adverse cardiovascular ischemic event; mono, monotherapy with rivaroxaban; and TP, thrombocytopenia.

Regarding the timing of bleeding complications, Figure 1A shows that the 2 curves begin to diverge at ≈1 year after enrollment, and the choice of antithrombotic regimens resulted in the differences in bleeding events within 3 years (Figure 2A). No significant difference was observed in the incidence of bleeding events in patients receiving monotherapy with rivaroxaban regardless of thrombocytopenia (5.7% in the thrombocytopenia group versus 3.2% in the nonthrombocytopenia group (HR, 2.0 [95% CI, 0.48–8.34], *P*=0.34). Conversely, the incidence of bleeding events was found to be significantly higher in the patients with thrombocytopenia receiving combination therapy with rivaroxaban and a single antiplatelet drug (14.3% in the thrombocytopenia group versus 5.0% in the nonthrombocytopenia group; HR, 3.18 [95% CI, 1.27–7.97], *P*=0.014).

The differences in the incidence of MACE between the thrombocytopenia and nonthrombocytopenia groups begin to diverge at ≈2 years after enrollment (Figure 1B). The differences in the incidence of MACE between the 2 antithrombotic regimens were similar to those of major bleeding (Figure 2B). Long‐term combination therapy with rivaroxaban and a single antiplatelet drug tended to increase risk of MACE in patients with AF with thrombocytopenia (14.3% versus 6.9%; HR, 2.30 [95% CI, 0.93–5.70], *P*=0.07). However, if the patients with AF received monotherapy with rivaroxaban, the incidence of MACE was not significantly different regardless of thrombocytopenia (8.6% in the thrombocytopenia group versus 5.4% in the nonthrombocytopenia group (HR, 1.83 [95% CI, 0.57–5.84], *P*=0.31).

### Results of the Multivariable Analysis

**Figure 3:**
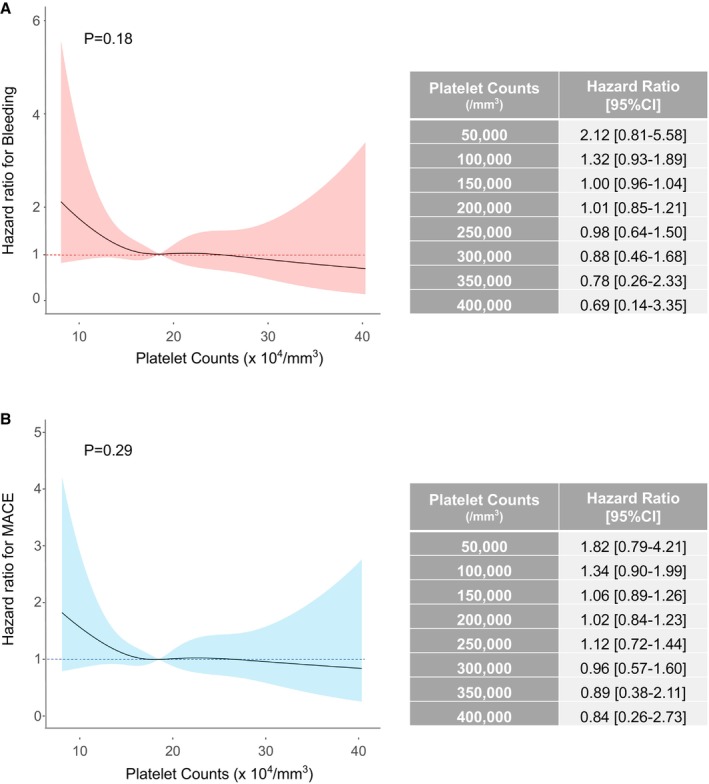
Association between platelet counts and major bleeding (A) or MACE (B). Restricted cubic spline curve shows the risks of major bleeding and MACE throughout the range of platelet counts. The table shows the values of hazard ratios with 95% CIs for the whole range of platelet counts at intervals of 50 000/mm^3^. MACE indicates major adverse cardiovascular ischemic event.

Table 3 shows that 4 factors were clinically relevant variables for major bleeding in the univariable analysis. In the Cox proportional hazards model, the occurrence of thrombocytopenia was identified as an independent predictor of major bleeding (HR, 2.57 [95% CI, 1.19–5.56], *P*=0.017) after being adjusted for age, hemoglobin value, and kidney function. The Fine–Gray model revealed a comparable result, indicating that the presence of thrombocytopenia was an independent predictor of major bleeding in patients with AF with CCS (HR, 2.43 [95% CI, 1.17–5.04]; *P*=0.017) (Table S4). Table 4 shows independent predictors of MACE, and widely known risk factors for atherosclerotic cardiovascular disease including chronic heart failure, old age, diabetes, and polyvascular disease were identified. However, thrombocytopenia was not associated with the occurrence of MACE. Regarding the incidence of major bleeding, no significant interaction was found between monotherapy with rivaroxaban and platelet counts (*P* for interaction=0.17) or MACE (*P* for interaction=0.48). A nonlinear relationship between platelet counts and adverse clinical outcomes was demonstrated by the restricted cubic spline curves, but a statistical significance was not reached (Figure 3A and 3B). Furthermore, the curves showed that the risks of major bleeding and MACE gradually increased at 150 000/mm^3^ and further escalated below 100 000/mm^3^.

**Table 3:** Cox Proportional Hazards Analyses for Major Bleeding

	HR [95% CI]	*P* value	Adjusted HR [95% CI]	*P* value
Thrombocytopenia	2.72 [1.26–5.88]	0.011	2.57 [1.19–5.56]	0.017
Age	1.05 [1.02–1.08]	0.001		
Hemoglobin	0.83 [0.74–0.93]	0.002		
eGFR	0.98 [0.97–0.99]	0.018		

eGFR indicates estimated glomerular filtration rate; and HR, hazard ratio.

**Table 4:** Cox Proportional Hazards Analyses for MACE

	HR [95% CI]	*P* value	Adjusted HR [95% CI]	*P* value
Age	1.05 [1.03–1.07]	<0.0001	1.04 [1.01–1.07]	0.001
Body mass index	0.93 [0.88–0.97]	0.003	0.94 [0.89–0.99]	0.018
Chronic heart failure	2.56 [1.82–3.60]	<0.0001	1.97 [1.38–2.82]	0.0002
Diabetes	1.52 [1.09–2.13]	0.015	1.72 [1.21–2.45]	0.0026
Polyvascular disease	1.69 [1.20–2.39]	0.003	1.48 [1.04–2.12]	0.03
Hemoglobin	0.88 [0.80–0.98]	0.01		
eGFR	0.98 [0.97–0.99]	<0.0001		
Thrombocytopenia	2.10 [1.03–4.29]	0.04		

eGFR indicates estimated glomerular filtration rate; HR, hazard ratio; and MACE, major adverse cardiovascular ischemic events.

## DISCUSSION

The major findings of this study demonstrated that the presence of baseline thrombocytopenia was significantly associated with an increased risk of major bleeding in the chronic phase. Moreover, even if a patient with AF with CCS is comorbid with thrombocytopenia, an appropriate antithrombotic drug could reduce major bleeding.

The BARC (Bleeding Academic Research Consortium) provided a standardized definition of bleeding end point for coronary stent trials, and baseline moderate and severe thrombocytopenia is considered 1 of 12 major high bleeding risk criteria.^15^ Despite the similarities between our findings and those of previous studies,^1^, ^2^, ^3^, ^15^ this study may still become a valuable addition to the limited evidence in the following points. First, all of the patients in this study were treated with rivaroxaban of direct oral anticoagulant due to AF, whereas the BARC criteria were determined based on the drug‐eluting stent studies, that is, antiplatelet‐based clinical trials. Practically, the ONXY‐ONE, ZEUS, and LEADERS‐FREE were randomized trials that directly compared second‐generation drug‐eluting stents, polymer‐free drug‐coated stents, and bare‐metal stents, respectively. These trials investigated the safety and efficacy of 1‐month short dual antiplatelet therapy in patients with high bleeding risk, but patients with AF treated with anticoagulant had a prevalence of 13% to 34%, and the number of patients with thrombocytopenia was quite low.^19^, ^20^, ^21^ Anticoagulant treatment is generally considered as a contraindication because managing patients with AF with thrombocytopenia is challenging due to the life‐threatening bleeding complications. The relationship between lower platelet counts and clinical outcomes were not assessed in 4 significant randomized trials that examined the efficacy and safety of direct oral anticoagulants for patients with AF, because those with baseline platelet count <100 000/mm^3^ or <90 000/mm^3^ were excluded.^4^, ^5^, ^6^, ^7^ According to the guidelines for treating thrombocytopenic patients with AF, treatment should be modified depending on platelet counts; for example, a combination therapy with a single antiplatelet agent and direct oral anticoagulant should be considered in patients with stable thrombocytopenia (platelet counts 75 000–10 000/mm^3^), and patients with <50 000/mm^3^ should be treated with a low dose of aspirin. However, evidence‐based recommendation evidence‐based recommendations are low grade because they are based on case reports and the results of low‐quality cohort studies, and the optimal duration of antithrombotic treatment is unclear.^13^ This study showed that a significantly higher incidence of major bleeding was observed in patients with AF with thrombocytopenia, and event survival curves showed that bleeding events of patients with AF with thrombocytopenia occurred after 1 year following enrollment in this study. When continuing antithrombotic therapy, patients and clinicians should have shared decision‐making regarding the possible bleeding event, especially in patients with severe thrombocytopenia.

Moreover, the study population included patients with AF with CCS in the chronic phase but not patients with acute coronary syndrome. Although the relationship between acquired acute thrombocytopenia and short‐term outcomes among patients with acute coronary syndrome was reported, it seems to be a temporary adverse effect because acquired acute thrombocytopenia may be associated with the use of unfractionated heparin, furosemide, nonsteroidal anti‐inflammatory drugs, and some antibiotics.^22^ Moreover, it could signify a higher degree of atherosclerosis predisposing to increased platelet consumption.^23^

Conversely, thrombocytopenia in this study may reflect a greater degree of comorbidities, including the elderly as well as high incidences of diabetes and heart failure. This study demonstrated that patients with AF with thrombocytopenia were associated with an increased risk of all‐cause mortality or a composite risk of cardiovascular death, myocardial infarction, or stroke compared with those without thrombocytopenia. The relationship between thrombocytopenia and ischemic events is still unclear, but several mechanisms would be involved, including patient vulnerability. An alternative explanation is that major bleeding and the onset of ischemic events are closely related. This study showed that the event curve of MACE among patients with AF with thrombocytopenia was increasing after ≈2 years following enrollment, and the timing was after major bleeding occurred. This finding is consistent with another subanalysis of the AFIRE trial, wherein patients with AF with CCS were more likely to have a major bleeding followed by a cardiovascular event or death. In a previous study, the risk of ischemic events increased nearly 8‐fold within 30 days after the development of major bleeding.^24^ Therefore, to minimize the risk of major bleeding, an ideal antithrombotic regimen should be established.

### Thrombocytopenia and Antithrombotic Regimens

Many studies have demonstrated that thrombocytopenia is strongly associated with an increased risk of bleeding complications and mortality and that changing the clinical scenario of poor prognosis is difficult.^1^, ^2^, ^3^, ^15^ In this regard, modifying antithrombotic regimens may improve prognosis, especially in reducing bleeding complications. This study demonstrated that the incidence of major bleeding within 3 years was significantly high in patients with AF with thrombocytopenia receiving a combination of rivaroxaban and a single antiplatelet agent. Although the finding of this study should be considered as a hypothesis generated due to post‐hoc analysis, de‐escalation from combination treatment to rivaroxaban monotherapy as soon as possible would be reasonable in patients with AF with CCS who developed thrombocytopenia. However, the safety of an earlier de‐escalation to rivaroxaban monotherapy within 1 year remains unclear, as AFIRE trial enrolled patients with AF with CCS more than 1 year after percutaneous coronary intervention. Additionally, when patients with AF with thrombocytopenia needed percutaneous coronary intervention, whether to use aspirin or P2Y_12_ receptor inhibitors as a single antiplatelet agent is debatable. Aspirin has been well known to cause upper gastrointestinal and intracranial bleeding, and recent meta‐analysis revealed a neutral treatment effect for mortality and myocardial infarction and a lower risk of major bleeding with an antithrombotic regimen without aspirin.^25^, ^26^ Thus, P2Y_12_ receptor inhibitors may be useful to prevent both major bleeding and ischemic events.^27^ In this context, future research is necessary to investigate the efficacy and safety of rivaroxaban monotherapy after percutaneous coronary intervention for patients with AF with CCS.

Before drawing conclusions, the study findings should be carefully interpreted. First, this is a post‐hoc analysis using source data derived from a randomized trial in which an analysis of platelet counts was not prespecified. Therefore, the different treatment interventions could affect the clinical results. Second, platelet count was not available in all patients with AF (96.3%), and the cause of thrombocytopenia has not been determined. Third, despite the large number of patients included, the number of patients with AF with thrombocytopenia was very low (3.3%) and may have low statistical power for the analysis regarding major bleeding and cardiovascular events. Finally, the types of antiplatelet agent were selected as per the physician's discretion, which may have affected the end points.

Therefore, thrombocytopenia is associated with a significant increase in the risk of major bleeding in patients with AF who developed CCS after 1 year of revascularization or in patients with angiographically confirmed coronary artery disease who do not require revascularization. Selecting antiplatelet and anticoagulant agents must be given special consideration for patients with thrombocytopenia continuing antithrombotic therapy. De‐escalation to rivaroxaban monotherapy may be beneficial in reducing the risk of major bleeding, although given the study limitation, well‐designed, randomized controlled trials are needed to establish this strategy.

## Sources of Funding

This study was supported by the Japan Cardiovascular Research Foundation based on a contract with Bayer Yakuhin, Ltd., who had no role in the trial design, data collection or analysis; trial result interpretations, or writing of the manuscript.

## Disclosures

Dr Iijima received a consulting fee from Bayer Yakuhin, Daiichi Sankyo, and Nippon Boehringer Ingelheim. Dr Nakamura received grants and personal fees from Bayer Yakuhin, Daiichi Sankyo, and Sanofi, and personal fees from Bristol‐Myers Squibb and Nippon Boehringer Ingelheim. Dr Yasuda received grants from Takeda Pharmaceutical, Abbott, and Boston Scientific, and personal fees from Daiichi‐Sankyo and Bristol‐Meyers. Dr Kaikita reports grants from Grants‐in‐Aid for Scientific Research (20K08451) from the Ministry of Education, Culture, Sports, Science and Technology of Japan, and grants and personal fees from Bayer Yakuhin and Daiichi Sankyo. Dr Akao received grants from the Japan Agency for Medical Research and Development, AMED, personal fees from Bristol‐Myers Squibb and Nippon Boehringer Ingelheim, and grants and personal fees from Bayer Yakuhin and Daiichi Sankyo. Dr Ako received personal fees from Bayer Yakuhin and Sanofi and grants and personal fees from Daiichi Sankyo. Dr Matoba received grants from the Japan Cardiovascular Research Foundation and personal fees from Nippon Boehringer Ingelheim, Daiichi Sankyo, AstraZeneca, and Bayer Yakuhin. Dr Miyauchi received personal fees from Amgen Astellas BioPharma, Astellas Pharma, MSD, Bayer Yakuhin, Sanofi, Takeda Pharmaceutical, Daiichi‐Sankyo, Nippon Boehringer Ingelheim, and Bristol‐Myers Squibb. Dr Hagiwara received grants and personal fees from Bayer Yakuhin, grants from Nippon Boehringer Ingelheim, and personal fees from Bristol‐Myers Squibb. Dr Kimura received grants from the Japan Cardiovascular Research Foundation, grants and personal fees from Bayer Yakuhin, Daiichi Sankyo, Sanofi, MSD, and AstraZeneca, and personal fees from Bristol‐Myers Squibb and Nippon Boehringer Ingelheim. Dr Hirayama received grants and personal fees from Boston Scientific Japan, Otsuka Pharmaceutical, Sanofi, Astellas Pharma, Bristol‐Myers Squibb, Daiichi Sankyo, and Bayer Yakuhin, grants from Fukuda Denshi, Abbott Japan, Japan, Takeda Lifeline, Sumitomo Dainippon Pharmaceutical, and personal fees from Toa Eiyo, Nippon Boehringer Ingelheim, Amgen Astellas BioPharma, and AstraZeneca. Dr Ogawa received personal fees from Towa Pharmaceutical, Bristol‐Meyers Squibb, Pfizer, Toa Eiyo, Bayer Yakuhin, and Novartis Pharma. The remaining authors have no disclosures to report.

## Supporting information

Data S1
Table S1–S4
Figure S1
