# Supplementary material for: Thrombocytopenia as a Bleeding Risk Factor in Atrial Fibrillation and Coronary Artery Disease: Insights From the AFIRE Study
Source: J Am Heart Assoc. 2023 Oct 17;12(20):e031096. doi: 10.1161/JAHA.123.031096 (PMC10757527; doi:10.1161/JAHA.123.031096)
Supplement: Supplementary file 1 — Data S1 Table S1–S4 Figure S1 [file JAH3-12-e031096-s001.pdf]

# SUPPLEMENTAL MATERIAL

**The AFIRE Investigators**


1. Satoshi Yasuda, M.D., Ph.D.
2. Koichi Kaikita, M.D., Ph.D.
3. Masaharu Akao, M.D., Ph.D.
4. Junya Ako, M.D., Ph.D.
5. Tetsuya Matoba, M.D., Ph.D.
6. Masato Nakamura, M.D., Ph.D.
7. Katsumi Miyauchi, M.D., Ph.D.
8. Nobuhisa Hagiwara, M.D., Ph.D.
9. Kazuo Kimura, M.D., Ph.D.
10. Atsushi Hirayama, M.D., Ph.D.
11. Kunihiko Matsui, M.D., M.P.H.
12. Hisao Ogawa, M.D., Ph.D.


**Departments and institutions**


1. Department of Cardiovascular Medicine, Tohoku University Graduate School of Medicine, 1-1 Seiryo-machi, Aoba-ku, Sendai 980-8574, Japan
2. Division of Cardiovascular Medicine and Nephrology, Department of Internal Medicine, Faculty of Medicine, University of Miyazaki, Miyazaki, Japan
3. Department of Cardiology, National Hospital Organization Kyoto Medical Center, 1-1 Mukaihata-cho, Fukakusa, Fushimi-ku, Kyoto 612-8555, Japan
4. Department of Cardiovascular Medicine, Kitasato University School of Medicine, 1-15-1 Kitasato, Minami-ku, Sagamihara 252-0373, Japan
5. Department of Cardiovascular Medicine, Faculty of Medical Sciences, Kyushu University, 3-1-1 Maidashi, Fukuoka 812-8582, Japan
6. Division of Cardiovascular Medicine, Toho University Ohashi Medical Center, 2-22-36, Ohashi, Meguro-ku, Tokyo 153-8515, Japan
7. Department of Cardiovascular Medicine Juntendo Tokyo Koto Geriatric Medical Center, 3-3-20 Shinsuna, Koto-ku, Tokyo 136-0075, Japan

8. Department of Cardiology, Tokyo Women's Medical University, 8-1, Kawada-cho, Shinjuku-ku, Tokyo 162-8666, Japan
9. Department of Cardiology, Yokosuka City Hospital, 1-3-2, Nagasaka, Yokosuka, 240-0195, Japan
10. Department of Cardiology Osaka Police Hospital, 10-31 Kitayama-cho, Tennouji-ku, Osaka City, Osaka 543-0035, Japan
11. Department of General Medicine, Kumamoto University Hospital, 1-1-1 Honjo, Chuo-ku, Kumamoto 860-8556, Japan
12. Kumamoto University, 2-39-1 Kurokami, Chuo-ku, Kumamoto 860-8555, Japan

**Table S1. Baseline characteristics of patients with or without major bleeding**

| | Major bleeding (+) n = 91 (%) | Major bleeding (−) n = 2042 (%) | p-value |
|---|---|---|---|
| Age, years | 76.9 ± 8.4 | 74.2 ± 8.2 | 0.003 |
| Age > 75 years | 55 (60.4) | 1067 (52.3) | 0.13 |
| Female sex | 18 (19.8) | 430 (21.1) | 0.90 |
| Body mass index | 24.5 ± 3.7 | 24.1 ± 3.4 | 0.36 |
| Body mass index < 18.5 kg/m$^2$ | 3 (3.3) | 73 (3.6) | 0.99 |
| Hypertension | 75 (82.4) | 1749 (85.7) | 0.36 |
| Dyslipidemia | 63 (69.2) | 1422 (69.6) | 0.91 |
| Diabetes mellitus | 42 (46.2) | 853 (41.8) | 0.45 |
| Current smoker | 13 (14.3) | 264 (12.9) | 0.75 |
| Chronic heart failure | 37 (40.7) | 725 (35.5) | 0.32 |
| e-GFR, mL/min/1.73m$^2$ | 54.4 ± 16.1 | 58.2 ± 16.3 | 0.03 |
| e-GFR < 60 mL/min/1.73m$^2$ | 59 (64.8) | 1122 (54.9) | 0.07 |
| Hemoglobin, g/dL | 13.0 ± 1.7 | 13.5 ± 1.7 | 0.003 |
| Hemoglobin < 11g/dL | 14 (15.4) | 163 (8.0) | 0.02 |
| Thrombocytopenia | 7 (7.7) | 63 (3.1) | 0.03 |
| Rivaroxaban monotherapy | 35 (38.5) | 1040 (50.9) | 0.02 |
| Prior myocardial infarction | 28 (30.8) | 716 (35.1) | 0.43 |

| | | | |
|---|---|---|---|
| Prior stroke | 16 (17.6) | 294 (14.4) | 0.37 |
| Prior PCI | 67 (73.6) | 1440 (70.5) | 0.56 |
| Prior CABG | 13 (14.3) | 226 (11.1) | 0.31 |
| Polyvascular disease | 45 (49.5) | 1009 (49.4) | 0.99 |
| Multivessel coronary disease | 18 (19.8) | 347 (17.0) | 0.48 |

Continuous variables are expressed as the mean ± SD. Categorical variables are expressed as n (percentage).

eGFR, estimated glomerular filtration rate; PCI, percutaneous coronary intervention;

CABG, coronary artery bypass graft surgery

**Table S2. Baseline characteristics of patients with or without MACE**

| | MACE (+) n = 135 (%) | MACE (−) n = 1998 (%) | p-value |
|---|---|---|---|
| Age, years | 77.0 ± 9.0 | 74.2 ± 8.1 | <0.0001 |
| Age > 75 years | 89 (65.9) | 1033 (51.7) | 0.001 |
| Female sex | 35 (25.9) | 413 (20.7) | 0.16 |
| Body mass index | 23.5 ± 4.0 | 24.5 ± 3.6 | 0.003 |
| Body mass index < 18.5 kg/m$^2$ | 13 (9.6) | 61 (3.1) | 0.001 |
| Hypertension | 115 (85.2) | 1709 (85.5) | 0.90 |
| Dyslipidemia | 89 (65.9) | 1396 (69.9) | 0.34 |
| Diabetes mellitus | 70 (51.9) | 825 (41.3) | 0.02 |
| Current smoker | 24 (17.8) | 253 (12.7) | 0.11 |
| Chronic heart failure | 78 (57.8) | 684 (34.2) | <0.0001 |
| e-GFR, mL/min/1.73m$^2$ | 53.0 ± 17.1 | 58.4 ± 16.2 | 0.0002 |
| e-GFR < 60 mL/min/1.73m$^2$ | 92 (68.1) | 1089 (54.5) | 0.002 |
| Hemoglobin, g/dL | 13.2 ± 1.9 | 13.5 ± 1.7 | 0.02 |
| Hemoglobin < 11g/dL | 17 (12.6) | 160 (8.0) | 0.07 |
| Thrombocytopenia | 8 (5.9) | 62 (3.1) | 0.08 |
| Rivaroxaban monotherapy | 59 (43.7) | 1016 (50.9) | 0.11 |
| Prior myocardial infarction | 54 (40) | 690 (34.5) | 0.23 |
| Prior stroke | 25 (18.5) | 285 (14.3) | 0.21 |
| Prior PCI | 95 (70.4) | 1412 (70.7) | 0.92 |

| | | | |
|---|---|---|---|
| Prior CABG | 18 (13.3) | 221 (11.1) | 0.40 |
| Polyvascular disease | 83 (61.5) | 971 (48.6) | 0.004 |
| Multivessel coronary disease | 25 (18.5) | 340 (17.0) | 0.64 |

Continuous variables are expressed as the mean ± SD. Categorical variables are expressed as n (percentage).

MACE, major adverse cardiac ischemic events; eGFR, estimated glomerular filtration rate;

PCI, percutaneous coronary intervention; CABG, coronary artery bypass graft surgery

**Table S3. Clinical outcomes of patients with platelet counts < 150,000/mm$^3$**

| | Platelet counts < 150,000/mm$^3$ n = 441 (%) | Platelet counts ≥150,000/mm$^3$ n = 1692 (%) | p-value |
|---|---|---|---|
| The primary endpoints | | | |
| Major bleeding | 25 (5.7) | 66 (3.9) | 0.11 |
| CV death + myocardial infarction + stroke | 30 (6.8) | 105 (6.2) | 0.66 |
| The secondary endpoints | | | |
| All cause death | 36 (8.2) | 76 (4.5) | 0.004 |
| CV death | 18 (4.1) | 50 (3.0) | 0.23 |
| Myocardial infarction | 3 (0.7) | 17 (1.0) | 0.78 |
| Stroke | 12 (2.7) | 51 (3.0) | 0.88 |

CV, cardiovascular

**Table S4. Fine-Gray hazard model for major bleeding**

| | HR [95%Cl] | p-value | Adjusted HR [95%Cl] | p-value |
|---|---|---|---|---|
| Thrombocytopenia | 2.60 [1.22-5.53] | 0.014 | 2.43 [1.17-5.04] | 0.017 |
| Age | 1.05 [1.01-1.08] | 0.006 | | |
| Hemoglobin | 0.84 [0.75-0.94] | 0.002 | | |
| eGFR | 0.98 [0.97-0.99] | 0.03 | | |

eGFR, estimated glomerular filtration rate;

HR, hazard ratio; 95%Cl: 95% confidence interval

**Figure S1 Distribution of baseline plateletcounts.**

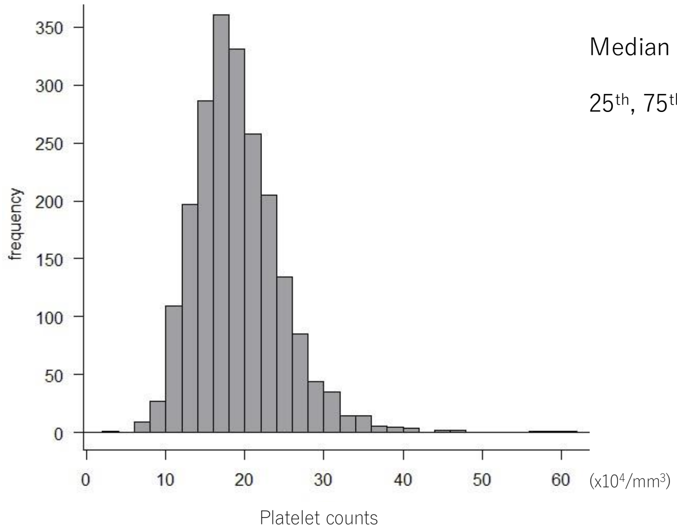


Median 18.5 x$10^4$/mm$^3$

25$^{th}$, 75$^{th}$ percentiles [15.6, 22.2]
